# Current Drug Treatment Strategies for Atrial Fibrillation and TASK-1 Inhibition as an Emerging Novel Therapy Option

**DOI:** 10.3389/fphar.2021.638445

**Published:** 2021-03-04

**Authors:** Manuel Kraft, Antonius Büscher, Felix Wiedmann, Yannick L’hoste, Walter E. Haefeli, Norbert Frey, Hugo A. Katus, Constanze Schmidt

**Affiliations:** ^1^ Department of Cardiology, University of Heidelberg, Heidelberg, Germany; ^2^ DZHK (German Center for Cardiovascular Research), Partner Site Heidelberg/Mannheim, University of Heidelberg, Heidelberg, Germany; ^3^ HCR, Heidelberg Center for Heart Rhythm Disorders, University of Heidelberg, Heidelberg, Germany; ^4^ Clinic for Cardiology II: Electrophysiology, University Hospital Münster, Münster, Germany; ^5^ Department of Clinical Pharmacology and Pharmacoepidemiology, University of Heidelberg, Heidelberg, Germany

**Keywords:** atrial fibrillation, anti-arrhythmic drugs, TASK-1, Cardioversion, KCNK3

## Abstract

Atrial fibrillation (AF) is the most common sustained arrhythmia with a prevalence of up to 4% and an upwards trend due to demographic changes. It is associated with an increase in mortality and stroke incidences. While stroke risk can be significantly reduced through anticoagulant therapy, adequate treatment of other AF related symptoms remains an unmet medical need in many cases. Two main treatment strategies are available: rate control that modulates ventricular heart rate and prevents tachymyopathy as well as rhythm control that aims to restore and sustain sinus rhythm. Rate control can be achieved through drugs or ablation of the atrioventricular node, rendering the patient pacemaker-dependent. For rhythm control electrical cardioversion and pharmacological cardioversion can be used. While electrical cardioversion requires fasting and sedation of the patient, antiarrhythmic drugs have other limitations. Most antiarrhythmic drugs carry a risk for pro-arrhythmic effects and are contraindicated in patients with structural heart diseases. Furthermore, catheter ablation of pulmonary veins can be performed with its risk of intraprocedural complications and varying success. In recent years TASK-1 has been introduced as a new target for AF therapy. Upregulation of TASK-1 in AF patients contributes to prolongation of the action potential duration. In a porcine model of AF, TASK-1 inhibition by gene therapy or pharmacological compounds induced cardioversion to sinus rhythm. The DOxapram Conversion TO Sinus rhythm (DOCTOS)-Trial will reveal whether doxapram, a potent TASK-1 inhibitor, can be used for acute cardioversion of persistent and paroxysmal AF in patients, potentially leading to a new treatment option for AF.

## 1 Introduction

Atrial fibrillation (AF) is the most common sustained cardiac arrhythmia with a prevalence of up to 4% in modern western populations and 0.5–2% in Asians (Fuster et al., 2006; Zoni-Berisso et al., 2014; Zulkifly et al., 2018), indicating the increased AF risk of Whites compared to Asians, Hispanics, and Blacks (Dewland et al., 2013). Differences in genetics, environmental exposures, cardiac structures, and ascertainment of AF are discussed as possible reasons but so far without a definite answer (Nazeri et al., 2010; Dewland et al., 2013; Gillott et al., 2017; Lee et al., 2017; Ugowe et al., 2018). AF is a predominant disease of the elderly population, with prevalence rates of 10.0–17.0% for patients over the age of 80 compared to 0.1% under the age of 49 (Zoni-Berisso et al., 2014). In 2010, the number of people worldwide suffering from AF was estimated to range around 33.5 million (Chugh et al., 2014) with an increased prevalence in children of parents with AF (Fox et al., 2004; Øyen et al., 2012). Furthermore, 30–40% of patients undergoing cardiac surgery and even 10–20% of patients with non-cardiac surgery develop new on-set AF (Chebbout et al., 2018; Dobrev et al., 2019). As triggers, a combination of inflammation, cardiac ischemia, altered atrial cytosolic calcium handling, and sympathetic activation are discussed, whereas the underlying mechanisms of postoperative AF are not yet fully understood (Aguilar and Nattel, 2019; Dobrev et al., 2019; Fakuade et al., 2020).

AF is associated with an increase of overall mortality of 1.5-fold in men and 2-fold in women (Benjamin et al., 1998; Stewart et al., 2002; Andersson et al., 2013). It is connected with an almost 5-fold increase in stroke incidences (Wolf et al., 1991) and about 20–30% of all ischemic stroke patients suffer from AF (Henriksson et al., 2012). Therefore, stroke prevention represents a central aspect of AF therapy (Hindricks et al., 2020). However, while death by stroke can be greatly reduced by anticoagulant therapy (Hart et al., 1999, 2007; Ruff et al., 2014; Piazza et al., 2020) there is still a residual stroke rate of 1.5% per year (Kirchhof et al., 2013). Furthermore, cardiovascular death remains common in patients suffering from AF (Kotecha et al., 2014) with a death rate of about 3.5% per year (Kirchhof et al., 2013).

In AF therapy, there are two predominant treatment strategies: rhythm control that aims to restore and sustain sinus rhythm (SR) and rate control that modulates ventricular heart rate and prevents development of tachymyopathy (Hindricks et al., 2020). In the existing literature, there are many clinical trials, systematic reviews, and meta-analyses slightly favoring one strategy above the other but, in cumulative analyses, the outcome of both treatment strategies seems to be comparable and to depend largely on comorbidities and severity of AF (de Denus et al., 2005; Chatterjee et al., 2013; Al-Khatib et al., 2014; Kotecha and Kirchhof, 2014; Nattel et al., 2014; Conkbayir et al., 2019; Conte et al., 2019; de Vecchis et al., 2019; Willems et al., 2019). Very recently, however, Kirchhof et al. (2020) showed in the Early Treatment of Atrial Fibrillation for Stroke Prevention Trial (EAST-AFNET 4), that rhythm control might be associated with a lower risk of death for cardiovascular reasons than rate control in patients with early (less than 1 year) AF, suggesting that rhythm control could be superior at least in this patient population.

Although many therapeutic options are available, there is still an unmet need in AF therapy due to insufficient efficacy and cardiac as well as extracardiac side effects of current treatments, which may negatively influence their therapeutic benefit (Ravens, 2010, 2017). To satisfy this unmet treatment need the underlying causes of AF have become a focus of research, leading to recognition of the tandem of P domains in a weak inward rectifying K^+^ channel (TWIK)-related acid sensitive K^+^ channel 1 (TASK-1), a member of the two-pore-domain potassium (K_2P_) channel family (Schmidt et al., 2015, 2017; Ravens, 2017). The atrial-selective TASK-1 current plays an important role in the repolarization phase of the atrial action potential (AP) (Schmidt et al., 2015, 2017; Schmidt and Peyronnet, 2018). AF induced changes in the TASK-1 current lead to an altered action potential duration (APD), which can help maintain AF, therefore, showing an involvement of TASK-1 in the pathophysiology of AF (Schmidt et al., 2015, 2017). Taken into account its atrial-selective expression in the human heart, its upregulation in AF, and its contribution to AF related APD shortening, TASK-1 represents a promising target for the treatment of AF (Schmidt et al., 2015, 2017).

In the following review we provide an overview of the existing literature regarding the two predominant treatment strategies for AF as well as the current state of research on TASK-1 in the context of AF therapy.

## 2 Rate Control Therapy

One of the two main treatment strategies for AF is rate control therapy. Modulation of the ventricular heart rate prevents development of tachymyopathy and is often enough to improve AF-related symptoms (Hindricks et al., 2020). First-line therapy in acute and long-term rate control are beta-blockers (Jones et al., 2014; Hindricks et al., 2020). In SR patients with heart failure they achieve a reduction in all-cause mortality which however, has recently been questioned in AF patients (Kotecha et al., 2014). Usually, they are well tolerated by all ages and have a potential for symptomatic and functional improvements (Kotecha et al., 2014, 2016).

Alternatively, or in combination, if the desired heart rate is not achieved with monotherapy, non-dihydropyridine calcium channel blockers can be used (Hindricks et al., 2020). However, if administered in combination with beta-blockers, a careful monitoring of the heart rate via 24 h electrocardiogram (ECG) to check for bradycardia should be done (Hindricks et al., 2020). Verapamil and diltiazem slow heart rate and are able to reduce symptoms of AF (Ulimoen et al., 2013). They are, however, not safe in use for patients with heart failure (Goldstein et al., 1991; Elkayam, 1998).

Another option for rate control therapy are cardiac glycosides (Hindricks et al., 2020). They act through an inhibition of the Na^+^–K^+^-ATPase (Schatzmann, 1953; Glynn, 1957; Schatzmann and Räss, 1965). They have been in use for over 200 years and are still controversially discussed (Goldberger and Alexander, 2014). The Digitalis Investigation Group (1997) could show in a randomized trial, that digoxin did not reduce mortality in patients with heart failure but reduced the rate of hospitalization. In this trial, however, patients with AF were excluded. Several studies have observed an increased mortality in AF patients taking digoxin (Hallberg et al., 2007; Whitbeck et al., 2013; Turakhia et al., 2014; Charnigo et al., 2018). Interpretation of the results from cardiac glycoside studies is often difficult as Ziff et al. (2015) found out in their meta-analysis that patients with a prescription of digoxin have generally more co-morbidities than patients without digoxin, potentially explaining a selection bias toward higher all-cause mortality in the digoxin group. Consequently, digoxin is still recommended by the new European Society of Cardiology (ESC) guideline for the management of AF (Hindricks et al., 2020). As several of the mentioned studies could link digoxin toxicity to elevated plasma levels a stringent dose adjustment is necessary, especially in patients with renal impairment. In conjunction with the topic of this review, a blockade of the TASK-1 current (I_TASK-1_) has been demonstrated by our group (Schmidt et al., 2018).

If rate control is not sufficient, the use of amiodarone can be considered. Because of its many extracardiac adverse effects (Vorperian et al., 1997; Jafari-Fesharaki and Scheinman, 1998; Cohen-Lehman et al., 2010; Papiris et al., 2010) it should represent a last resort for rate control therapy.

The optimal target heart rate for AF patients is not clearly defined. Van Gelder et al. (2010) found in the Rate Control Efficacy in Permanent Atrial Fibrillation (RACE) II study that lenient rate control with a resting heart rate of less than 110 bpm is as effective as a strict rate control with a heart rate of less than 80 bpm at rest and less than 110 bpm during moderate exercise in regard to death through cardiovascular reasons, hospitalization for stroke, heart failure, systemic embolism, bleeding, and life-threatening arrhythmic events, at least for patients with preserved left ventricular ejection fraction. Furthermore, the lenient rate control was easier to achieve and non-inferior in the endpoint of quality of life (Van Gelder et al., 2010; Groenveld et al., 2011).

In case of an unsuccessful therapy with pharmacological rate control, an ablation of the atrioventricular (AV) node or His bundle may be performed. The overall mortality for this method is low (Queiroga et al., 2003) and quality of life improves (Lim et al., 2007). Of course, after ablation of the AV node, patients remain pacemaker-dependent for the rest of their life. To prevent tachymyopathy a biventricular pacing device should be preferred in patients with reduced left ventricular ejection fraction. Therefore, AV node ablation should only be considered after a failed pharmacological approach or in patients who already have a pacemaker implanted. It further represents a worthwhile option to improve the percentage of biventricular pacing in cardiac resynchronization therapy (CRT) patients with AF.

## 3 Rhythm Control Therapy

Rhythm control therapy is the second main treatment strategy for AF, aiming to restore and sustain SR. Cardioversion from AF to SR can be achieved by pharmacological or electrical means (Shea and Maisel, 2002). To help maintain SR, antiarrhythmic drugs (AADs), catheter ablation, or surgical ablation (if the patient undergoes heart surgery for coronary artery bypass graft or valve replacement) are available (Hindricks et al., 2020). Furthermore, for recent-onset AF in hemodynamically stable patients, Pluymaekers et al. (2019) showed in the Rate Control vs. Electrical Cardioversion Trial 7—Acute Cardioversion vs. Wait and See (RACE 7 ACWAS) that a wait-and-see approach was non-inferior to early cardioversion in achieving SR after 4 weeks. Early cardioversion has been defined as pharmacological cardioversion (pCV) as soon as possible after presentation at the emergency department, whereas, delayed cardioversion comprised solely of rate control therapy for the first 48 h followed by cardioversion if needed. As they observed spontaneous conversion in 69% of patients in the delayed cardioversion group, no further treatment was needed for most patients.

However, while the results from the RACE 7 ACWAS trial are in support of a wait-and-see approach as a further therapy option for recent-onset AF, there is still some debate about the merits of this approach (Botto and Tortora, 2020; Capucci and Compagnucci, 2020). Capucci and Compagnucci (2020) argued against wait-and-see and give for consideration that quality of life assessment, performed after 4 weeks, does not show the initial dissatisfaction of the patients in the delayed group after discharge from the emergency department. On the other hand, this may not be the case as in many patients rate control therapy is sufficient enough for symptom relief and therefore improvement of quality of life (Botto and Tortora, 2020). Another point is patient safety, as the risk for AF complications increases with duration and some studies indicate that longer AF episodes promote the inducibility and stability of AF (Capucci and Compagnucci, 2020). In contrast, however, while active cardioversion is generally considered as safe it still has several side effects and therefore the question remains if immediate treatment is acceptable for a condition that is likely to resolve spontaneously (Botto and Tortora, 2020). In the end, the choice will also depend on practical feasibility as for the wait-and-see approach access to prompt follow-up is necessary (Botto and Tortora, 2020; Capucci and Compagnucci, 2020).

### 3.1 Electrical Cardioversion

Zoll et al. (1956) performed the first successful external electrical cardioversion (eCV) of ventricular fibrillation in four human patients in 1955 and around 10 years later Lown et al. (1963) described eCV of AF in 50 patients. In eCV a synchronized electrical current is applied to the heart to restore SR. However, while it is an effective method (Mittal et al., 2000; Kirchhof et al., 2002, 2005; Morani et al., 2019), it requires sedation of the patient with, e.g., benzodiazepines or propofol (Notarstefano et al., 2007; Furniss and Sneyd, 2015; Morani et al., 2019) and therefore, normally a fasting period of 6 h before eCV is needed (Nattel and Opie, 2006). Furthermore, administration of oxygen and its control with pulse oximetry as well as an ECG monitoring are required. As a result, cardioversion has to be performed in a hospital or at least a similar setting in the presence of trained professionals (Furniss and Sneyd, 2015).

About 30% of patients have another AF episode within a three-month follow-up (De Simone et al., 2003; Loricchio et al., 2007; Xia et al., 2009). Some studies showed even higher recurrence rates of up to 43% after 30 days (Malouf et al., 2005; Zarauza et al., 2006). Therefore, many patients need more than one eCV (Botkin et al., 2003; Raitt et al., 2006). Potential side effects of this procedure are, in addition to the anesthetic risk, skin burns, heart block, prolonged sinus arrest, ventricular pro-arrhythmia, epidermal necrosis, and apnea (Pagan-Carlo et al., 1997; Kowey et al., 1998; Yava et al., 2012). However, while most studies show no relevant myocardial damage from external eCV (Georges et al., 1996; Neumayr et al., 1997; Greaves and Crake, 1998; Walcott et al., 2003; Piechota et al., 2007; Lobo et al., 2018), there is injury to the myocardium by internal eCV (Noro et al., 2016; Stieger et al., 2018).

### 3.2 Pharmacological Cardioversion

In 1918 Walter Frey proposed the pharmacological active substance quinidine for the conversion of AF to SR (Frey, 1918). Since then, many substances were tested for their anti-arrhythmic potency so that today several AADs are available for pCV (amiodarone, disopyramide, dofetilide, dronedarone, esmolol, flecainide, ibutilide, procainamide, propafenone, quinidine, sotalol, and vernakalant; see also Supplementary Table S1 for a full list of recommended AADs) (Boriani et al., 2004; Marinelli and Capucci, 2010; Schilling, 2010; January et al., 2014; Hindricks et al., 2020). Compared to eCV, pCV is not as successful in restoring SR (Chen et al., 2013) and is connected with longer hospitalization (Dankner et al., 2009; Cristoni et al., 2011; Bellone et al., 2012; Gitt et al., 2013; Crijns et al., 2014). However, because the patients do not need to be sedated, no fasting is required (Hindricks et al., 2020). The substances recommended by the ESC for acute pCV are summarized in Table 1. Another possible drug for acute cardioversion is dofetilide, which is not available in Europe (European Medicines Agency, 2004). Each drug comes with its own drawbacks and limitations (Hindricks et al., 2020).

**TABLE 1:** Overview of recommended antiarrhythmic drugs for acute pharmacological cardioversion by the ESC with their rate and time of cardioversion, targets and class after Vaughan Williams.

	Cardioversion			
Drug	Rate (%)	Time	Channels	Class
Amiodarone	5–85	6–18 h	I_Na_, I_Ca_, I_K_, I_KNa_, I_KACh_, I_TASK-1_	III
Flecainide	52–92	25–52 min	I_Na_	Ic
Ibutilide	43–77	26–54 min	I_Na_, I_Kr_	III
Propafenone	49–85	29–287 min	I_Na_	Ic
Vernakalant	47–93	8–16 min	I_Na_, I_Kur_, I_KACh_, I_Kr_, I_to_	III

In 1970 Miles Vaughan Williams proposed a system of four classes for the characterization of AADs after their mode of action (Vaughan Williams, 1984). The first class are sodium channel inhibitors which are divided in three subgroups according to their effect on APD and the strength of the channel block (Campbell and Vaughan Williams, 1983). Class Ia produces a moderate block and increased APD, class Ib a weak block and reduced APD, and class Ic a marked block and conserved APD (Lei et al., 2018). Class II drugs are comprised of β-blocker and reduce sino-atrial node pacing rates as well as slow AV node AP conduction (Dukes and Vaughan Williams, 1984). Class III drugs are potassium channel blockers that slow AP repolarization and prolong the effective refractory period (Lei et al., 2018). Lastly, Class IV drugs are calcium channel blockers which reduce heart rate and conduction, especially on the AV node and sino-atrial node (Lei et al., 2018).

### 3.2.1 Class Ic AADs

Flecainide and propafenone block fast sodium channel currents (I_Na_) and belong to the class Ic AADs after Vaughan Williams (Cowan and Vaughan Williams, 1981; Vaughan Williams, 1984; Harrison, 1985; Roden and Woosley, 1986; Funck-Brentano et al., 1990; Wang et al., 1992). Flecainide has been shown to be successful in cardioversion of recent-onset AF in 52–92% of cases depending on the observation period. The mean time to cardioversion ranged from 110 to 190 min after oral administration and 25–52 min after intravenous infusion (Madrid et al., 1993; Reisinger et al., 1998; Alp et al., 2000; Martínez-Marcos et al., 2000; Khan, 2003; Reisinger et al., 2004; Balla et al., 2011; Bash et al., 2012). Flecainide has been shown to be superior to propafenone and amiodarone in both time and rate of cardioversion (Martínez-Marcos et al., 2000; Balla et al., 2011), similar to ibutilide (Reisinger et al., 2004), and inferior to vernakalant (Pohjantähti-Maaroos et al., 2019). The most common adverse events during a therapy with flecainide are dizziness (30%), visual disturbance (28%), headache (9%), and nausea (9%). Pro-arrhythmia has been observed in 7–8% of patients (Holmes and Heel, 1985).

Propafenone has a cardioversion rate of 49–85% in recent-onset AF, depending on the observation time point and the route of administration, and a mean time of cardioversion of 29–30 min after intravenous and 166–287 min after oral administration (Capucci et al., 1999; Madonia et al., 2000; Martínez-Marcos et al., 2000; Khan, 2001; Zhang et al., 2005; Balla et al., 2011; Conde et al., 2013). Studies comparing propafenone to other drugs showed an inferiority to vernakalant in both time and rate of cardioversion (Conde et al., 2013) as well as flecainide (Martínez-Marcos et al., 2000; Balla et al., 2011), to ibutilide in rate with a non-significant inferiority in time (Zhang et al., 2005) and superiority to amiodarone in rate and time (Martínez-Marcos et al., 2000; Balla et al., 2011). Adverse events observed under the treatment of propafenone mostly affect the cardiovascular, gastrointestinal and nervous system. The most common adverse effects are dizziness (12.5%), nausea/vomiting (10.7%), unusual taste (8.8%), constipation (7.2%), and fatigue (6.0%). The most frequent cardiac adverse events are pro-arrhythmia (4.7%), angina (4.6%), congestive heart failure (3.7%), and ventricular tachycardia (3.4%) (Capucci and Boriani, 1995).

Propafenone and flecainide should not be used in patients with structural heart diseases as they are associated with a high pro-arrhythmic risk and increase mortality in these cases as demonstrated for flecainide in the Cardiac Arrhythmia Suppression Trial (CAST) (Morganroth, 1987; Echt et al., 1991; Podrid and Anderson, 1996; Andrikopoulos et al., 2015; Tamargo et al., 2015; Hindricks et al., 2020). Furthermore, in most trials structural heart diseases have been an exclusion criterion (Khan, 2003).

### 3.2.2 Class III AADs

Amiodarone is the original representative of class III AADs as it leads to prolongation of the APD (Vaughan Williams, 1984; Harrison, 1985). It has a cardioversion rate for recent-onset AF of about 5–85% and a mean time to cardioversion of 6–18 h after intravenous application (Noc et al., 1990; Joseph and Ward, 2000; Martínez-Marcos et al., 2000; Kafkas et al., 2007; Balla et al., 2011; Camm et al., 2011; Savelieva et al., 2013). In studies directly comparing amiodarone against flecainide and propafenone as well as vernakalant it was inferior in both time and rate of cardioversion (Martínez-Marcos et al., 2000; Balla et al., 2011; Camm et al., 2011). Compared to ibutilide it has been inferior in time but the difference in rate has not been statistically significant (Kafkas et al., 2007). However, it is important to note that especially in studies comparing amiodarone against faster acting AADs the window of observation has an impact on the outcome. Some studies stopped after 90 min whereas the effect of amiodarone takes a longer time, resulting in low cardioversion rates of amiodarone compared to studies with a longer observation period (Martínez-Marcos et al., 2000; Balla et al., 2011; Camm et al., 2011).

Amiodarone is a multi-channel blocker, inhibiting inward sodium (I_Na_) and calcium channel currents (I_Ca_) as well as voltage- and ligand-gated potassium channel currents (I_K_, I_KNa_, I_KACh_) (Kodama et al., 1999) and the two-pore domain potassium channel TASK-1 (I_TASK-1_) (Gierten et al., 2010). There are numerous side effects described in the literature, whereas hypothyroidism is the most common with a rate of up to 22%. (Amiodarone Trials Meta-Analysis Investigators, 1997; Kodama et al., 1999; Goldschlager et al., 2007). Another serious adverse effect is pulmonary toxicity (Wolkove and Baltzan, 2009) with dose-dependent incidence rates of about 2–6% (Dusman et al., 1990; Goldschlager et al., 2007). Other side effects are a blue discoloration and photosensitivity of the skin, photophobia, halo visions as well as optic neuropathy, gastrointestinal side effects, bradycardia, AV-block, and pro-arrhythmia (Goldschlager et al., 2007; Camm and Savelieva, 2008). However, most of these side effects are mainly observed during prolonged therapy and not in the setting of acute cardioversion (Singh, 1996; Wolkove and Baltzan, 2009).

To reduce the side effects of amiodarone, the substance has been modified (Nattel and Singh, 1999). Removal of the two iodine atoms and the addition of a methanesulfonyl group results in dronedarone, which has similar electrophysiological effects as amiodarone (Sun et al., 1999, 2002). It is a multi-channel blocker acting on potassium (I_Kr_, I_Ks_, I_K1_, I_KACh_, I_TASK-1_, I_SUS_), sodium (I_Na_), calcium (I_Ca_), and hyperpolarization-activated cyclic nucleotide-gated channels (I_f_) (Thomas et al., 2003; Kathofer et al., 2005; Bogdan et al., 2011; Schweizer et al., 2011; Schmidt et al., 2012).

In the ATHENA trial (A placebo-controlled, double-blind, parallel arm Trial to assess the efficacy of dronedarone 400 mg bid for the prevention of cardiovascular Hospitalization or death from any cause in patiENts with Atrial fibrillation/atrial flutter) a decrease in hospitalization due cardiovascular events or death in patients with AF that are treated with dronedarone has been found (Hohnloser et al., 2008, 2009). However, contrary to the expectations the PALLAS (Permanent Atrial Fibrillation Outcome Study Using Dronedarone on Top of Standard Therapy) trial had to be stopped for safety reasons because patients with permanent AF in risk of major vascular events had an increased rate of heart failure, stroke, and death of cardiovascular reasons under treatment with dronedarone compared to placebo (Connolly et al., 2011). It also increased mortality in patients with symptomatic heart failure and severe left ventricular systolic dysfunction (Køber et al., 2008). Today, dronedarone is not indicated for the use in acute cardioversion of AF (European Medicines Agency, 2019).

Further class III AADs are the methanesulfonamide derivatives ibutilide and dofetilide (Murray, 1998; Mounsey and DiMarco, 2000). Ibutilide prolongs the APD through blockade of the rapid component of cardiac delayed rectifier potassium currents (I_Kr_) and the activation of slow delayed inward sodium currents (I_Na_) (Murray, 1998; Glatter et al., 2001). It is associated with a risk for torsades de pointes of about 5% (Kowey et al., 1996; Nattel and Singh, 1999; Gowda et al., 2003; Nair et al., 2011). The cardioversion rate of ibutilide in recent-onset AF is reported to be around 43–77% and the mean time to cardioversion after intravenous infusion 26–54 min (Gowda et al., 2003; Kafkas et al., 2007; Simon et al., 2017; Vogiatzis et al., 2017). Kafkas et al. (2007) showed in their study that ibutilide is superior in cardioversion time to amiodarone with no significant difference in cardioversion rate and Zhang et al. (2005) showed a superiority to propafenone in cardioversion rate but not time. It is similar to flecainide (Reisinger et al., 2004) and inferior to vernakalant (Simon et al., 2017; Vogiatzis et al., 2017) in time and rate of cardioversion.

Dofetilide inhibits the rapid component of the delayed rectifier potassium current (I_Kr_) (Mounsey and DiMarco, 2000; Roukoz and Saliba, 2007). Similar to ibutilide it has a risk for torsades de pointes of 0.3–10.5% (Falk et al., 1997; Nørgaard et al., 1999; Bianconi et al., 2000; Lenz and Hilleman, 2000). The rate of cardioversion is between 12.5 and 53% depending on drug dose and the duration of AF and the mean time to cardioversion is 40–55 min (Suttorp et al., 1992; Sedgwick et al., 1995; Falk et al., 1997; Nørgaard et al., 1999; Bianconi et al., 2000). It is safe for patients after myocardial infarction as well as congestive heart failure and left ventricular systolic dysfunctions (Shenasa and Shenasa, 2016).

Vernakalant is effective in conversion of recent-onset AF with a rate of 47–70% in the first 90 min after intravenous infusion, in small, non-randomized trials up to 93%. The median time to cardioversion is around 8–16 min (Roy et al., 2004, 2008; Kowey et al., 2009; Camm et al., 2011; Bash et al., 2012; Conde et al., 2013; Savelieva et al., 2013; Torp-Pedersen et al., 2013; Carbajosa et al., 2017; Simon et al., 2017; Vogiatzis et al., 2017; Kossaify, 2019; Pohjantähti-Maaroos et al., 2019). It is superior to ibutilide (Simon et al., 2017; Vogiatzis et al., 2017), propafenone (Conde et al., 2013), amiodarone (Camm et al., 2011) and flecainide (Pohjantähti-Maaroos et al., 2019) in both cardioversion rate and time to cardioversion. However, the effect on AF with a duration of more than seven days is reduced (Torp-Pedersen et al., 2013).

Vernakalant blocks sodium currents (I_Na_) in a frequency and voltage-dependent manner. At normal heart rates and membrane resting potentials the inhibition is low but increases with faster rates and less negative potentials as seen in the atria during AF compared to the ventricle. Furthermore, vernakalant blocks atrial-specific ultra-rapid delayed rectifier potassium currents (I_Kur_), acetylcholine-activated atrial potassium currents (I_KACh_) and transient outward potassium currents (I_to_). An inhibition of the rapid component of cardiac delayed rectifier potassium currents (I_Kr_) has been observed *in vitro* but this effect seems to be too small to have therapeutic relevance. Taken together, this leads to an almost atrial-selective effect with no ventricular pro-arrhythmic effects (Fedida et al., 2005; Fedida, 2007). Serious adverse effects of vernakalant are hypotension and AV-block (Kowey et al., 2009; Savelieva et al., 2013; Torp-Pedersen et al., 2013). It is safe in use for patients with a history of ischemic heart disease as long as they have no hypotension or severe aortic stenosis (Savelieva et al., 2013; Torp-Pedersen et al., 2013) but the risk for adverse effects may be higher in patients with heart failure (Savelieva et al., 2013).

### 3.3 Catheter Ablation

An alternative to drug treatment is catheter ablation (Hindricks et al., 2020). This method is based on Haissaguerre et al. (1998) who described triggers for paroxysmal AF in the pulmonary veins and the termination of AF through their ablation by radio-frequency. Pulmonary vein isolation (PVI) is superior to AADs in maintaining SR with a comparable rate of complications (Cosedis Nielsen et al., 2012; Mont et al., 2014). However, only about 40% of patients are free from AF after a six year follow-up period and about 60% needed more than one procedure (Sorgente et al., 2012). Isolation of pulmonary veins can be achieved via radio frequency ablation or cryoballoon ablation. Kuck et al. (2016) compared both methods and found that cryo-ablation is non-inferior to radio-frequency ablation. However, the primary safety endpoint of death, cerebrovascular events, and serious treatment-related adverse events has been reached in over 10% of patients in both ablation groups (Kuck et al., 2016). To test if catheter ablation is superior to pharmacological drug therapy, the Catheter Ablation vs. Anti-arrhythmic Drug Therapy for Atrial Fibrillation (CABANA) trial has been initiated. The primary endpoint of the study was total mortality, disabling stroke, serious bleeding, or cardiac arrest (Packer et al., 2018). The trial shows no significant reduction of this endpoint with catheter ablation compared to anti-arrhythmic drug therapy. However, major limitations of this trial are a lower than expected event rate and treatment crossovers that require consideration when interpreting the results (Packer et al., 2019).

A similar trial but with a narrower patient population is the Catheter Ablation vs. Standard Conventional Treatment in Patients with Left Ventricular Dysfunction and Atrial Fibrillation (CASTLE-AF) trial that compared catheter ablation to pharmacological standard therapy in patients with AF and left ventricular dysfunction. The primary endpoint was the composite of all-cause mortality or worsening of heart failure requiring unplanned hospitalization (Marrouche and Brachmann, 2009). The results of the trial are a significant reduction in all-cause mortality and rate of hospitalizations for worsening of heart failure in the ablation group (13.4%) compared to the medication group (25.0%) (Marrouche et al., 2018). This trial, however, comes with a couple of possible pitfalls such as (I) missing data on the quality of rate control in patients in the medication group, (II) the late appearing effect in the all-cause mortality where only about 50% of the patients remained in the follow-up, (III) significant differences in patient characteristics and, (IV) the fact that some sub-groups like patients including an ejection fraction of less than 25% did not appear to have a benefit from ablation. Furthermore, all patients had symptomatic AF with previously failed AAD therapy or were intolerant or unwilling to take AADs (Bono and Kirchhof, 2018).

Catheter ablation has potential periprocedural complications such as pericarditis, pericardial effusion, cardiac tamponade, stroke, pulmonary vein stenosis, severe esophageal injury leading to atrioesophageal fistula, phrenicus lesions, and pacemaker dependency (Sacher et al., 2006; Cosedis Nielsen et al., 2012; Mont et al., 2014; Han et al., 2017; Kapur et al., 2017; Yetter et al., 2017; Hindricks et al., 2020). These severe adverse events occur in up to 7% of patients and up to 3% will even have life-threatening complication whereof less than 0.2% die (Dagres et al., 2009; Cappato et al., 2010; Gupta et al., 2013).

Electrical and pharmacological cardioversion as well as catheter ablation all have their own drawbacks and limitations as mentioned above. Especially the risk of potentially life-threatening ventricular arrhythmia is a significant limitation for the use of AADs. Therefore, there is a need for new potential targets and drugs for the treatment of AF (Ravens, 2010; Schmidt et al., 2011). In this regard the identification of atrial selective drug targets would be essential to overcome ventricular side effects of AAD therapy (Peyronnet and Ravens, 2019). In the following, this review will focus on the TASK-1 channel, a potassium channel that shows atrial specific expression and is associated with a characteristic electrical remodeling of atrial tissue in AF (Schmidt et al., 2015).

## 4 Electrical Remodeling

Electrical remodeling is part of the cardiac response to functional and structural stressors with the initial aim of maintaining and compensating cardiac performance (Cutler et al., 2011). However, over time, this compensation mechanism turns maladaptive and leads to arrhythmia and pump failure (Cutler et al., 2011). This process could be shown in the atrium of goats with artificially induced AF resulting in a shortening of the atrial effective refractory period (AERP), a reversion of its physiological rate adaptation, and an increase in rate, inducibility and stability of AF (Wijffels et al., 1995). For this phenomena Wijffels et al. (1995) coined the phrase “Atrial fibrillation begets atrial fibrillation” as they could show that AF induced remodeling leads to a progression of AF.

The alterations in cardiac electrical function summarized as electrical remodeling are the subject of current research and not yet completely understood (Nattel et al., 2020). Altered calcium handling through the reduction of the L-type calcium channel current (I_CaL_) has been observed (Christ et al., 2004) as well as alterations in potassium channel currents, for example upregulation of the inward-rectifier potassium channel K_ir_2.1 and therefore increase of I_K1_ (Gaborit et al., 2005), decrease of the transient outward potassium current I_to_ (Nattel et al., 2007), suppression of the ultrarapid delayed rectifier current I_Kur_ (Nattel et al., 2008), an increase of the slow delayed rectifier current I_Ks_ (Caballero et al., 2010), and a decreased expression of Small-conductance Ca^2+^-activated K^+^ (SK) channels (Yu et al., 2012). Beside changes in ion-channel expression there are also alterations in other cellular structures. For example, the expression of gap junction protein Conexin43 is suppressed in AF (Bikou et al., 2011).

To describe all the molecular alterations that take place in electrical remodeling during AF would be beyond the scope of this review. Therefore, we will focus on TASK-1, a newly identified potassium channel that is associated with a typical upregulation during AF.

## 5 Two-Pore-Domain Potassium Channels

Hodgkin and Huxley already proposed the presence of leak currents in 1952 (Hodgkin and Huxley, 1952) but it took another 40 years to identify the K_2P_ channels as being responsible for this instantaneous and non-inactivating current, making them the newest identified group of potassium channels (Duprat et al., 1997; Lesage and Lazdunski, 2000; Goldstein et al., 2001, 2005). Until today, the channel family comprises 15 members with a common structure element of two pore-forming (P) loops and four transmembrane domains (M) per subunit (Ketchum et al., 1995; Goldstein et al., 2005). On the cell membrane they assemble as hetero- and homodimers and are regulated by a variety of different stimuli such as signaling lipids, temperature, pressure, pH-level, and a number of pharmaceutical substances (Lesage and Lazdunski, 2000; Goldstein et al., 2001; Lesage, 2003; Dedman et al., 2009; Feliciangeli et al., 2015). However, they are relatively insensitive to typical potassium channel blockers such as tetraethylammonium, 4-aminopyridine, cesium, and barium ions (Lesage, 2003). The different members of the family show either a weak inward (Lesage et al., 1996), open (Duprat et al., 1997), or outward rectification (Maingret et al., 2002).

K_2P_ channels are involved in ion homeostasis, hormone secretion, cell development, and excitability. Therefore, these channels play a role in many physiological and pathological mechanism such as vascular and pulmonary hypertension, cardiac arrhythmia, nociception, neuroprotection, taste and temperature sensing, anesthesia, apoptosis, carcinogenesis, and depression (Patel and Honore, 2001; Bayliss and Barrett, 2008; Enyedi and Czirják, 2010; Lloyd et al., 2011; Williams et al., 2013; Feliciangeli et al., 2015; Kim and Kang, 2015).

## 5.1 TASK-1 in Atrial Fibrillation

TASK-1 is a member of the K_2P_ channel family and represents a promising target for AF therapy (Limberg et al., 2011; Schmidt et al., 2015, 2017). It is predominantly expressed in the human atria (Schmidt et al., 2015, 2017). Higher mRNA expression levels in the left atrium (14-fold) and right atrium (16-fold) of humans compared to human left ventricular tissue samples were measured (Schmidt et al., 2015). Furthermore, it was shown that atrial tissue undergoes an electrical remodeling process during AF—indicating an atrial cardiomyopathy—that leads to a strong upregulation of TASK-1 mRNA and protein levels, which are observed on a functional level as well (Barth et al., 2005; Schotten et al., 2011; Schmidt et al., 2015).

Furthermore, the current density of TASK-1 in isolated human atrial cardiomyocytes was 3 times higher in patients with chronic AF compared to SR. In paroxysmal AF patients, an increase by 1.5-fold was observed (Schmidt et al., 2015). Inhibition of TASK-1 currents in isolated human atrial cardiomyocytes of patients with chronic AF leads to a prolongation of the APD of up to 30% (Schmidt et al., 2015). In combination of these two facts it could be shown that an upregulation of TASK-1 leads to a shortening of the APD in patients with chronic AF (Schmidt et al., 2015, 2017). This could be verified by the application of A293, a specific TASK-1 inhibitor. Whereas, A293 had almost no effect on the APD of atrial cardiomyocytes from patients with SR or paroxysmal AF, it has been able to prolong it in patients with chronic AF to a duration very similar to SR (Schmidt et al., 2015). Taken together, the TASK-1 current participates predominantly in the atrial APD shortening of patients with AF. This property indicates a promising new anti-arrhythmic target to inhibit AF.

These experimental findings have been verified in a computational model of a human atrial cardiomyocyte. For this purpose, the model from Grandi et al. (2011) has been modified to include the TASK-1 currents. The model also reproduced that shortening of the APD in chronic AF through higher expression of TASK-1 has pro-arrhythmic effects (Schmidt et al., 2015, 2017). In contrast to AF patients, a downregulation of TASK-1 has been observed in patients with severe impairment of left ventricular function, leading to the assumption that a therapy with TASK-1 inhibitors would have a smaller effect on patients with paroxysmal AF and severely impaired left ventricular function compared to patients with a normal left ventricular function (Schmidt et al., 2015, 2017).

In a porcine animal model it could be shown that pigs with artificially induced AF via atrial burst stimulation by an implanted pacemaker using a biofeedback pacing algorithm developed similar upregulation of TASK-1 and shortening of the APD as seen in human patients. Pigs were treated with an adeno-associated viral vector carrying anti–TASK-1-siRNA which was injected in both atria and led to a reduced expression of TASK-1. This treatment led to a prolongation of the APD and reduction of TASK-1 current in the siRNA group compared to AF control pigs and to a significant reduction in AF burden (Schmidt et al., 2019b).

Liang et al. (2014) showed that loss-of-function mutations of TASK-1 lead to increased susceptibility to develop AF in the presence of pro-arrhythmic stimuli and Harleton et al. (2015, 2013) observed a phosphorylation dependent TASK-1 dysregulation in a canine model of AF, further underlining its role in AF.

While these data clearly show the role of TASK-1 in the pathophysiology of AF, this is not the case for the other TASK channels (Rinné et al., 2015; Schmidt et al., 2015). For example, tandem of P domains in a weak inward rectifying K^+^ channel (TWIK)-related acid sensitive K^+^ channel 3 (TASK-3) that has long been known as a non-cardiac channel (Putzke et al., 2007; Schmidt et al., 2015), has been found in expression analysis of human atrial auricles (Rinné et al., 2015). However, in patch-clamp experiments, performed on isolated rat cardiomyocytes, no functional evidence for TASK-3 currents could be obtained (Putzke et al., 2007). Rinné et al. (2015) confirmed these results in human cardiomyocytes, finding only TASK-1 currents and intermediate currents between TASK-1 and TASK-3, concluding that TASK-3 forms heterodimers with TASK-1 in human cardiomyocytes. Compared to TASK-1 the cardiac expression of TASK-3 was shown to be substantially lower, therefore, the role of TASK-3 in the atrial AP remains to be seen (Rinné et al., 2015; Schmidt et al., 2015). Finally, for the other TASK channels there is no data available showing an involvement in the atrial AP, suggesting that they are unsuitable targets for AF therapy (Gurney and Manoury, 2009; Schmidt and Peyronnet, 2018; Peyronnet and Ravens, 2019).

Beside TASK-1, other atrial-selective channels have been proposed as possible drug targets in the therapy of AF (Ravens, 2017). The first such candidate was the voltage-gated outward-rectifying K^+^ channel Kv1.5 (Fedida et al., 1993). Courtemanche et al. (1999) predicted a prolongation of the atrial APD through inhibition of Kv1.5 in a mathematical AF model. However, experimental validation led to mixed results depending strongly on AF induced electrical remodeling (Wettwer et al., 2004; Ravens, 2017; Schmidt and Peyronnet, 2018). In human SR samples an inhibition of Kv1.5 shortened the atrial APD, possibly resulting in the maintenance of reentrant excitation (Loose et al., 2014). Contrary, in AF samples a prolongation of the atrial APD was observed upon Kv1.5 inhibition (Loose et al., 2014). However, since Kv1.5 is—in contrast to TASK-1— downregulated in chronic AF patients, the possible therapeutic effect of Kv1.5 inhibition is in question (Van Wagoner et al., 1997; Schmidt and Peyronnet, 2018). Therefore, in accordance with these data, no clinical studies that show a definite reduction of AF burden have been reported (Ravens, 2017; Schmidt and Peyronnet, 2018).

Another group of channels discussed in the context of atrial-selectivity are the SK channels (Ravens, 2017). However, while SK 1 and SK 2 seem to be more abundant expressed in the atria, SK 3 is uniformly expressed in the atrium and ventricle, question if SK channels are really atrial-selective (Tuteja et al., 2005). Furthermore, the role of SK channels in modifying the shape of the atrial AP in AF patients remains far from clear as both, AF-related up- and downregulation have been observed (Skibsbye et al., 2014; Ravens, 2017; Schmidt and Peyronnet, 2018). Therefore, while inhibition of SK channels is expected to prolong the APD and consecutively protect against re-entry (Diness et al., 2010), pro-arrhythmic events have also been observed (Hsueh et al., 2013). Taken together, these data question the possible usage of SK channel blockers in AF therapy (Ravens, 2017; Schmidt and Peyronnet, 2018).

In conclusion, the TASK-1 channel represents a promising target for future therapy of AF since it is atrial-specific expressed, which reduces the risk of pro-arrhythmic effects in the ventricle, and has a strong influence on atrial APD in AF patients (Ravens, 2010; Schmidt et al., 2014b, 2015).

## 5.2 TASK-1 in Pulmonary Hypertension

Besides the heart, the TASK-1 channel is expressed in the liver, placenta, prostate, kidney, lung, pancreas, ovary, small intestine, and brain (Lesage and Lazdunski, 2000; Williams et al., 2013). Therefore, it is involved in a variety of physiological and pathophysiological processes. For example, TASK-1 plays a role in pulmonary hypertension (PH). PH is defined as an elevation of the mean pulmonary arterial pressure of more than 20 mmHg and a pulmonary vascular resistance greater or equal 3 Wood (Simonneau et al., 2019). It is a progressive disease that leads to right ventricular failure and ultimately to death (Humbert et al., 2004; Gaine and McLaughlin, 2017) with a 3-years survival of 68% (Hurdman et al., 2012). PH is caused by pathological remodeling of the pulmonary arteries, primarily through the excessive proliferation of pulmonary arterial smooth muscle cells (PASMCs). This leads to vasoconstriction in conjunction with an increase of pulmonary vascular resistance, followed by destruction of small-resistance pulmonary arteries and arterioles, and ends in right ventricular failure (Crosswhite and Sun, 2014; Thompson and Lawrie, 2017).

Potassium channels play an important role in PH, as decreased potassium channel activity in PASMCs increases resistance to apoptosis, cell proliferation and vasoconstriction leading to vascular remodeling (Boucherat et al., 2015; Olschewski et al., 2017). Olschewski et al. (2006) showed in isolated primary PASMCs that a knockdown of TASK-1 leads to depolarization of the cells and therefore to a higher pulmonary muscle tone. Ma et al. (2013) described six heterozygous miss-sense mutations of TASK-1, leading to loss of function, causing PH in patients. Navas Tejedor et al. (2017) found two homozygous mutations of TASK-1 in a Spanish patient cohort associated with a severe and early form of PH. Cunningham et al. (2019) could show that these two mutations lead to severely reduced TASK-1 channel current compared to wild type. While the guanylate cyclase activator, riociguat, a novel treatment for PH, enhances TASK-1 wild type channel current, it had no effect on the mutated channels (Cunningham et al., 2019).

Whether pharmacological inhibition of TASK-1 can also lead to PH is still not finally clarified (Olschewski et al., 2016; Schmidt et al., 2016; Wiedmann et al., 2021). Olschewski et al. (2016) give for consideration that endothelin-1, a strong endogenous inhibitor of the TASK-1 channel, can induce PH as well as dasatinib, a src tyrosine kinase inhibitor that inactivates the TASK-1 channel (Tang et al., 2009; Montani et al., 2012; Seyler et al., 2012; Nagaraj et al., 2013). As these data were not validated in *in vivo* animal models or human studies, the net effect of TASK-1 current on pulmonary muscle tone was largely unknown (Schmidt et al., 2016). Very recently, however, our group published some preliminary data on the hemodynamic effect of TASK-1 inhibition in a pig model. A mild increase in pulmonary artery pressure was observed upon acute TASK-1 inhibition (Wiedmann et al., 2021). Therefore, this potential side effect has to be considered in the development of TASK-1 inhibitors for future treatment of AF (Schmidt et al., 2016).

## 5.3 TASK-1 in Sleep Apnea

In addition to the involvement of TASK-1 in the pathophysiology of AF and PH, it also plays a role in sleep apnea. Sleep apnea is defined as pauses of breathing for more than 10 s with a stop of the airflow at nose and mouth of more than five times per hour and a partial neurological arousal (Guilleminault et al., 1976; Costanzo et al., 2015). There are three types of sleep apnea, obstructive sleep apnea (OSA), which originates in the obstruction of the upper airways (Laratta et al., 2017), central sleep apnea (CSA) with a temporary withdrawal of the brainstem-driven respiratory drive resulting in a stop of the activity of the respiratory muscle and therefore the airflow (Costanzo et al., 2015), and a mixed form of both (Gastaut et al., 1966).

OSA has a prevalence of 46% in the German population over the age of 20 years, whereas, only 6% suffer from OSA syndrome (Fietze et al., 2019). Most common symptoms are daytime sleepiness, unrefreshing sleep, and fatigue (Laratta et al., 2017). OSA is connected with an increased risk of myocardial infarction, congestive heart failure, coronary revascularization procedures, and cardiovascular death (Redline et al., 2010; Hla et al., 2015). Furthermore it is associated with insulin resistance, therapy-resistant hypertension and AF (Bradley and Floras, 2003; Somers et al., 2008; Digby and Baranchuk, 2012; Hohl et al., 2014; Jullian-Desayes et al., 2015).

CSA has a high prevalence in patients with heart failure of about 30–40% (Javaheri, 2006; Oldenburg et al., 2007; Macdonald et al., 2008). The symptoms of CSA are similar to OSA with insomnia, fatigue, and sometimes daytime sleepiness (Costanzo et al., 2015). One of the risk factors for CSA is AF besides male sex, lower ejection fraction, waking hypocapnia, higher New York Heart Association functional class, and higher B-type natriuretic peptide levels (Javaheri et al., 1998; Sin et al., 1999; Oldenburg et al., 2007; Calvin et al., 2011; Costanzo et al., 2015).

The detection of pH and blood gas is an essential part of the feedback control required for spontaneous breathing (Feldman et al., 2003). The sensing of O_2_ occurs in the carotid bodies in the periphery whereas CO_2_ is mainly measured by central chemoreceptors in the brainstem (Richerson, 1995; Nattie, 1999; Ballantyne and Scheid, 2000; Feldman et al., 2003). The CO_2_ central chemoreceptor (CCR) has a high sensitivity for the partial pressure of carbon dioxide (pCO_2_), as a rise of only 1 mmHg in pCO_2_ increases ventilation by 20–30% (Feldman et al., 2003). So far there has been no evidence of a direct sensing of CO_2_; in contrast CO_2_ sensing occurs through measuring of the pH-level (Feldman et al., 2003; Jiang et al., 2005). There are many proteins sensitive to changes in pH, for example low resistance gap junctions (Dean et al., 2001, 2002; Solomon et al., 2001; Solomon and Dean, 2002), inward rectifier potassium channels (Zhu et al., 2000; Jiang et al., 2001), pH-sensitive membrane ion-transport proteins (Putnam, 2001; Wiemann and Bingmann, 2001), and TASK channels (Bayliss et al., 2001; Washburn et al., 2002). Inhibition of TASK-1 by extracellular acidosis leads to depolarization of the membrane and increases excitability of the cell (Wang et al., 2008). Furthermore, Mulkey et al. (2004) could show that neither inwardly rectifying nor calcium-activated potassium channels but background potassium channels are responsible for the chemosensitivity of cells in the retrotrapezoid nucleus of rats. In addition, it has been shown by Patel et al. (1999) that halothane and isoflurane, two volatile anesthetics, activate TASK channel current. Furthermore, Takahashi et al. (2005) observed that halothane reduces the response to hypoxia, as hypoxia leads to an inhibition of TASK-channel currents, further suggesting a role of TASK in the chemosensing of the carotid body. However, Mulkey et al. (2007) suggested a few years later, that there had to be some other potassium channel, beside TASK-channels, responsible for the central respiratory chemosensitivity. While the involvement of TASK-1 may not be critical for the central respiratory chemosensitivity, it appears to be required for the pH sensing by the carotid body (Trapp et al., 2008; Guyenet et al., 2010). Nevertheless, an involvement of TASK-1 in ventilatory control via modulation of the respiratory output reacting to chemical stimuli seems likely (Wang et al., 2008).

OSA as well as CSA result from an unstable chemical control system, therefore leading to instability of the ventilation control (Hudgel et al., 1998; Younes et al., 2001; Wang et al., 2008). Wang et al. (2008) could show that TASK-1 plays a role in spontaneous sleep apnea in rats. They found a positive relation between TASK-1 protein content in the brainstem and the spontaneous sleep apnea index. They suggested an elevation of TASK-1 expression in the brainstem may lead to an increased occurrence of sleep apnea due to unstable respiratory control. This might be due to an augmented sensitivity of the CCR via heightened TASK-1 expression, resulting in an over-sensing of pCO_2_ changes, leading to an over-correction of the negative-feedback-loop and therefore to sleep apnea or other breathing disorders (Khoo et al., 1982; Khoo, 2000; Wang et al., 2008).

However, TASK-1 does not only assemble as homodimers but also as heterodimers with TASK-3. They share about 62% of their amino acid sequence (Rajan et al., 2000; Berg et al., 2004). Therefore, there still is some debate about whether only TASK-1 and heterodimers of TASK-1 and TASK-3 are involved in the breathing regulation, as it seems to be the case in rodents (Trapp et al., 2008; Wang et al., 2008) or if homodimers of TASK-3 are involved as was recently suggested by Cunningham et al. (2019).

Because the TASK-1 channel is involved in many pathological processes, a modulation of the channel by drugs could be a promising strategy against AF, sleep apnea or drug-induced ventilatory depression, sleep disorders, neurodegenerative disorders, major depression, and/or malignancy (Chokshi et al., 2015; Kiper et al., 2015; Wiedmann et al., 2016).

## 5.4 Inhibitors of TASK-1

Many inhibitors of TASK-1 have been originally developed as inhibitors of the voltage-gated potassium channel Kv1.5 (Kiper et al., 2015). Kv1.5 has been proposed as a promising target for the therapy of AF because it is expressed in the atrium but not the ventricle and its inhibition prolongs the atrial AP but does not influence ventricular repolarization (Decher et al., 2004, 2006). It plays a critical role in the AP repolarization of human atrial myocytes as it contributes to the delayed rectifier sustained outward current (I_Ksus_) (Fedida et al., 1993; Wang et al., 1993). In this context TASK-1 has been discussed as a target for AF as it contributes to I_Ksus_ as well, even though to a lesser extent than Kv1.5 (Limberg et al., 2011; Kiper et al., 2015).

Furthermore, it could be shown that the Kv1.5 blocker AVE0118 is effective against sleep apnea in a pig model of upper airway collapse induced through negative pressure (Wirth et al., 2013). However, it has been found that the Kv1.5 blockers A1899 (also known as S20951), A293 (also known as A1231), and S9947 are much more potent TASK-1 inhibitors (Knobloch et al., 2002, 2004; Putzke et al., 2007; Streit et al., 2011).

TASK-1 binds its inhibitors highly selective, with strong affinity and extremely low washout rates. Recently, Rödström et al. (2020) have been able to solve the structure of human TASK-1 by X-ray crystallography. They could show that high-affinity inhibitors are trapped in a vestibule below the selectivity filter by an X-gate, leading to the above mentioned low washout rates (Rödström et al., 2020). The entrapment has to be considered when interpreting data from pharmacokinetic-pharmacodynamic studies as inhibitors will remain bound and active for a longer time than predicted by these studies.

Given the role of TASK-1 in AF, it is not surprising that both AVE0118 as well as A293 among others have been reported as being effective against AF in animal models (Knobloch et al., 2002, 2004; Wirth et al., 2007). (For a complete list of all TASK-1 inhibitors tested in animal or human studies for their possible use in AF treatment see Table 2.) It has been shown that the porcine variant of TASK-1 is pharmacologically and functionally very similar to the human variant as they share about 97% of their identity as well as their selective expression in the atria (Schmidt et al., 2014a; Wiedmann et al., 2020). Therefore, our group tested A293 in a porcine model of paroxysmal AF. In this model AF was induced via right atrial burst pacing by implanted pacemakers. We could show in patch-clamp experiments on isolated cardiomyocytes from pigs with paroxysmal AF that inhibition of TASK-1 via application of A293 leads to prolongation of the APD. Furthermore, we observed that intravenous administration of A293 led to a significant prolongation of the atrial effective refractive period. We have also been able to restore sinus rhythm after elicitation of AF episodes by application of A293 with cardioversion times of about 3 min (Wiedmann et al., 2020). Similar results could be observed in an animal model of persistent AF (Wiedmann et al., 2021). To sum up, blockage of TASK-1 currents by A293 leads to anti-arrhythmic effects and acute cardioversion *in vivo* (Wiedmann et al., 2020, 2021).

**TABLE 2:** List of TASK-1 inhibitors that have been tested in animal or human studies for their possible use in atrial fibrillation treatment. For IC_50_ value measurement the humanTASK-1 channel has been heterogeneously expressed in *Xenopus laevis* oocytes (XO), chinese hamster ovarian cells (CHO) or human embryonic kidney cells (tsA201). For the IC_50_ measurements of doxapram in *X. leavis* oocytes ratTASK-1 has been used, no measurements with hu manTASK-1 in oocytes are published.

Substance	Cellular studies	Animal studies	Clinical studies
A1899	IC_50_ 0.04 μM (XO)^1^	AERP prolongation^2,3^	Phase I—unpublished^9^
(S20951)	IC_50_ 0.01 μM (CHO)^1^	Reduced left atrial vulnerability^2,3^	
A293	IC_50_ 0.22 μM (XO)^4^	Reduction of arrhythmia burden^6,7^	
(AVE1231)	IC_50_ 0.25 μM (XO)^5^	Acute pCV^6,7^	
		Rhythm control of persistent AF^6,7^	
		AERP prolongation^6–8^	
AVE0118	IC_50_ 0.603 μM (XO)^9^	AERP prolongation^3^	Phase IIa - unpublished^10^
		Reduced left atrial vulnerability^3^	
Doxapram	IC_50_ 0.41 μM (XO)^11^	Reduction of arrhythmia burden^13^	Phase Ib/IIa - ongoing (DOCTOS)^14^
	IC_50_ 4.0 μM (tsA201)^12^	Acute pCV^13^	
		Rhythm control of persistent AF^13^	
		AERP prolongation^13^	
S9947	IC_50_ 0.2 μM (XO)^9^	AERP prolongation^2,3^	
		Reduced left atrial vulnerability^2,3^	

Atrial effective refractory period (AERP); atrial fibrillation (AF); Doxapram Conversion To Sinus rhythm (DOTOS); pharmacological cardioversion (pCV).

^1^ Streit et al. (2011). ^2^ Knobloch et al. (2002). ^3^ Knobloch et al. (2004). ^4^ Putzke et al. (2007). ^5^ Schmidt et al. (2015). ^6^ Wiedmann et al. (2020). ^7^ Wiedmann et al. (2021). ^8^ Wirth et al. (2007). ^9^ Kiper et al. (2015). ^10^ Kambayashi et al. (2020). ^11^ Cotten et al. (2006). ^12^ Cunningham et al. (2019). ^13^ Schmidt et al. (2019a). ^14^ Peyronnet and Ravens (2019).

## 5.4.1 Doxapram

Another potent TASK-1 inhibitor is doxapram (Cotten et al., 2006). It has been developed in the 1960s for use in humans and animals as a drug to stimulate breathing and activate the carotid body (Lunsford et al., 1964). Doxapram is used as a breathing stimulant in patients with moderate to severe ventilatory failure to reverse hypercapnia and hypoxemia (Edwards and Leszczynski, 1967; Yost, 2006) as well as in patients with chronic obstructive pulmonary disease (Moser et al., 1973). In a clinical case study doxapram has been proposed as treatment for OSA (Houser and Schlueter, 1978; Yost, 2006). Furthermore, doxapram arouses patients after anesthesia and drug-induced central nervous system depression because of barbiturates, volatile anesthetics, nitrous oxide, and benzodiazepines (Evers et al., 1965; Riddell and Robertson, 1978; Rappolt Sr et al., 1980; Yost, 2006) and has been used for the treatment of post-operative shivering (Singh et al., 1993). Another indication for doxapram is apnea in newborns (Henderson-Smart and Steer, 2004; Mathew, 2011).

Administration of doxapram leads to an elevation of tidal volume as well as a marginal increase of respiratory rate (Winnie and Collins, 1966). Doxapram acts on different parts of the respiratory control system in a concentration-dependent manner. It modulates peripheral as well as central sites (Yost, 2006). Nishino et al. (1982) showed that doxapram acts on peripheral chemoreceptors in the carotid body. After sectioning the carotid sinus and aortic nerve in cats the ventilatory effect of doxapram has been abolished for doses as high as 6 mg/kg (Mitchell and Herbert, 1975; Nishino et al., 1982). Some residual ventilatory effect has been observed after denervation of peripheral chemoreceptors with even higher doses of doxapram, leading to the assumption of a secondary site of action probably in the central nervous system (Mitchell and Herbert, 1975; Wilkinson et al., 2010; Golder et al., 2013).

Doxapram is a very efficient TASK inhibitor with a half-maximal effective concentration of 410 nM for ratTASK-1, 37 μM for ratTASK-3 and 9 μM for a heterodimer of both in *Xenopus laevis* oocytes (Cotten et al., 2006). These values are within the range of therapeutic doxapram concentrations; therefore, it is plausible to assume that TASK-1 and TASK-3 present in glomus cells of the carotid body are mediating the ventilatory effects of doxapram (Yost, 2006; O’Donohoe et al., 2018). Inhibition of TASK-1 and TASK-3 depolarizes the membrane, followed by calcium influx through voltage-gated calcium channels, leading to secretion of neurotransmitters. These neurotransmitters cause excitation of afferent nerves terminating in respiratory and cardiovascular control centers in the brainstem, resolving respiratory failure induced by narcotics or central nervous system depressants (Buckler, 2007; Peers et al., 2010; Golder et al., 2013; O’Donohoe et al., 2018).

Interestingly, Cunningham et al. (2020) could show recently that doxapram inhibits both humanTASK-1 and humanTASK-3 equipotently (4.0 μM for TASK-1 vs. 2.5 μM for TASK-3), suggesting a possible reason for the differences observed between rodent models and humans. Furthermore, they could show that the two enantiomers of the racemate doxapram have different effects on TASK channels. Whereas, the (+)-enantiomer (GAL-054) has a higher inhibitory effect on humanTASK-1 (1.6 μM) and humanTASK-3 (1.4 μM), the effect of the (–)-enantiomer (GAL-053) is much reduced with 336 μM (humanTASK-1) and 286 μM (humanTASK-3) (Cunningham et al., 2020). Furthermore, Golder et al. (2012a, b) showed in rats that the adverse events such as arrhythmias, seizures, and death were only observed with GAL-053, whereas, the respiratory stimulant properties were connected with administration of GAL-054. However, GAL-054 leads to hypertension in rats with an increase in blood pressure of 15–20% above baseline (Golder et al., 2013).

Today, doxapram is rarely applied because of the introduction of safer and shorter-acting anesthetic drugs that have no need for an arousing breathing stimulant drug. Even its application in patients with respiratory insufficiency is almost obsolete as there are other substances and techniques available (Yost, 2006). However, in recent years there has been work by our group to investigate doxapram as a new drug for rhythm control in AF and help this substance to experience a renaissance.

Similar to A293, we administered doxapram in a porcine model for paroxysmal and persistent AF to test its efficacy in restoring and sustaining SR. The induction of AF was achieved by intermittent atrial burst pacing via implanted pacemakers. An AV-node ablation was performed to prevent tachymyopathy and an integrated biofeedback-algorithm prevented pacing during episodes of AF to promote the development of endogenous AF. A significant reduction of AF-burden has been observed in pigs under daily doxapram treatment. Furthermore, doxapram could be applied for cardioversion of paroxysmal AF in pigs (Schmidt et al., 2019a). Taken together, doxapram appears to be a promising drug for the treatment of AF.

However, being data from a large-animal model, these findings need to be verified in a human trial. Therefore, the DOxapram Conversion TO Sinus rhythm (DOCTOS)-Trial (EudraCT No: 2018-002979-17) has been initiated (Peyronnet and Ravens, 2019). Only patients with paroxysmal or persistent, non-valvular AF will be included in this study. The primary aim is to investigate the anti-arrhythmic potential of doxapram for acute cardioversion from AF to SR after intravenous application. Secondary aims are finding the ideal dose for cardioversion, to measure the time to cardioversion as well as the time to recurrence of AF within 7 days of cardioversion, to determine the doxapram pharmacokinetics after one or two equal consecutive bolus doses and lastly to gather data on the safety and tolerability of doxapram (EU Clinical Trials Register, 2018). A positive outcome of the DOCTOS-Trial would be a further step toward doxapram as a possible new treatment option for AF patients.

## 6 Conclusion

To conclude this review, there are many different options available to treat patients with AF, but none of them is without limitations and drawbacks. Therefore, it is essential to look for further ways to treat these patients. Underlying atrial cardiomyopathy mechanisms have to be elucidated to decide the best therapeutic strategy for each patient. A new option could become the inhibition of the TASK-1 potassium channel. Therefore, we are awaiting with interest the results of the DOCTOS trial and to answer the question: Is doxapram a new treatment option for AF? However, more scientific insights in AF are the key to develop successful and personalized treatment strategies for patients.

## Abbreviation:

AAD, antiarrhythmic drug; AERP, atrial effective refractory period; AF, atrial fibrillation; AP, action potential; APD, action potential duration; AV, atrioventricular; CCR, CO_2_ central chemoreceptor; CRT, cardiac resynchronization therapy; CSA, central sleep apnea; ECG, electrocardiogram; eCV, electrical cardioversion; ESC, European Society of Cardiology; K_2P_, two-pore-domain potassium; OSA, obstructive sleep apnea; PASMC, pulmonary arterial smooth muscle cell; pCO_2_, partial pressure of carbon dioxide; pCV, pharmacological cardioversion; PH, pulmonary hypertension; PVI, pulmonary vein isolation; SK, Small-conductance Ca^2+^-activated K^+^; SR, sinus rhythm; TASK-1, tandem of P domains in a weak inward rectifying K^+^ channel (TWIK)-related acid sensitive K^+^ channel 1; TASK-3, tandem of P domains in a weak inward rectifying K^+^ channel (TWIK)-related acid sensitive K^+^ channel 3
