# Supplementary material for: Current Drug Treatment Strategies for Atrial Fibrillation and TASK-1 Inhibition as an Emerging Novel Therapy Option
Source: Front Pharmacol. 2021 Mar 4;12:638445. doi: 10.3389/fphar.2021.638445 (PMC8058608; doi:10.3389/fphar.2021.638445)
Supplement: Supplementary file 1 [file table1.pdf]

frontiers

Supplementary Material

1 SUPPLEMENTARY TABLES

Table S1: List of antiarrhythmic drugs recommended by either the ESC or AHA/ACC/HRS for the treatment of atrial fibrillation.

Class	Drug	ESC[1]	USA[2]	Chemical structure
Ia	Disopyramide	no	yes	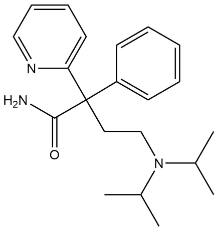
Ia	Procainamide	no	yes	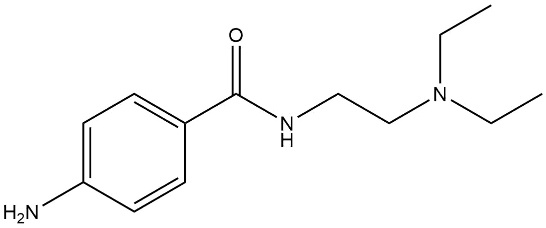
Ia	Quinidine	no	yes	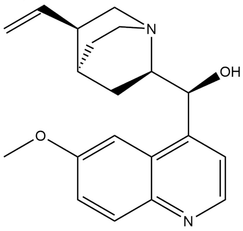
Ic	Flecainide	yes	yes	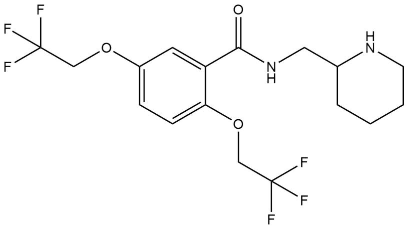
Ic	Propafenone	yes	yes	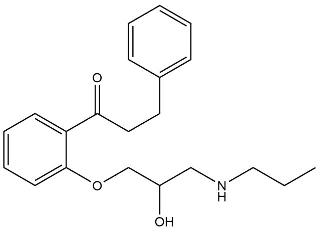
II	Atenolol	yes	yes	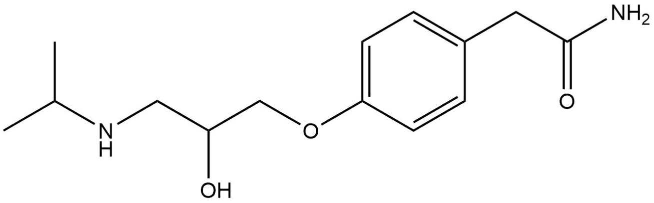

Class	Drug	ESC[1]	USA[2]	Chemical structure
II	Bisoprolol	yes	yes	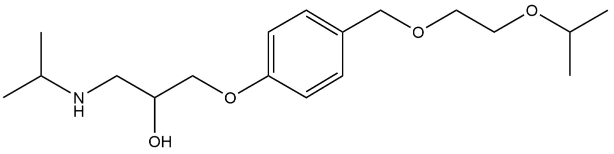
II	Carvedilol	yes	yes	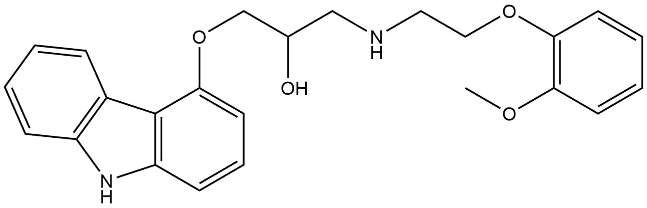
II	Esmolol	yes	yes	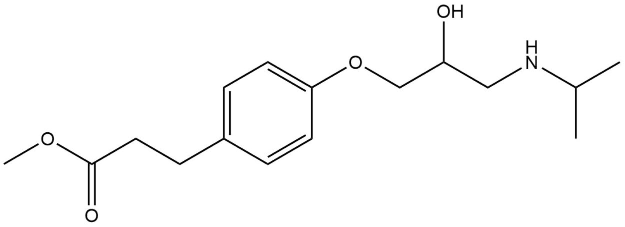
II	Landiolol	yes	-[3]	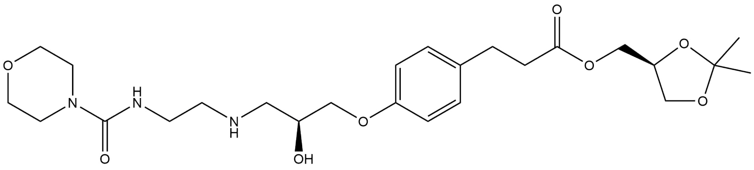
II	Metoprolol	yes	yes	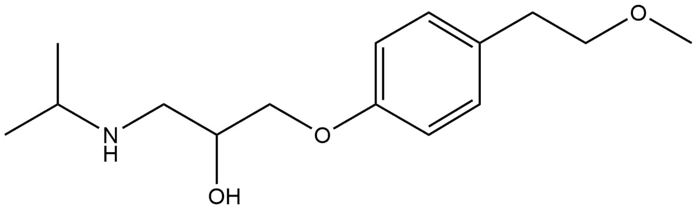
II	Nadolol	-[3]	yes	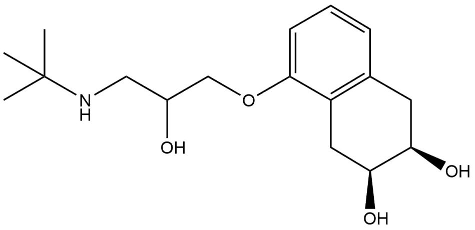
II	Nebivolol	yes	-[3]	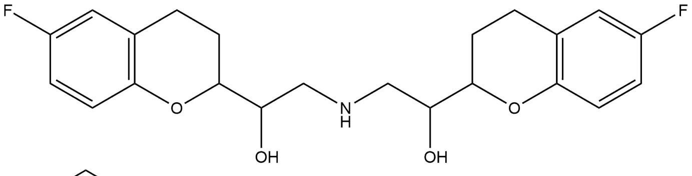
II	Propranolol	no	yes	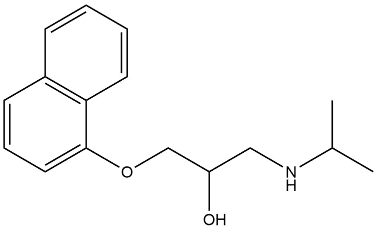

Class	Drug	ESC[1]	USA[2]	Chemical structure
III	Amiodarone	yes	yes	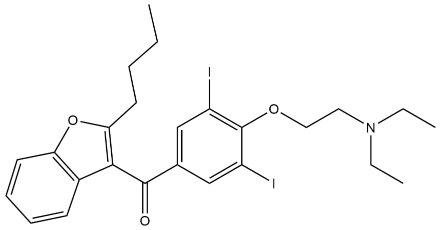
III	Dofetilide	yes	yes	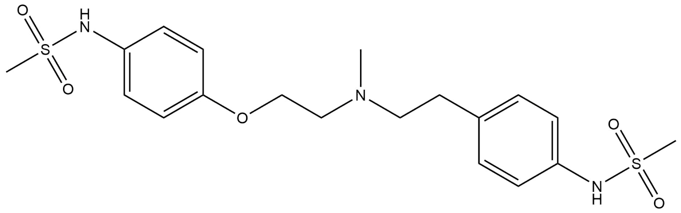
III	Dronedarone	yes	yes	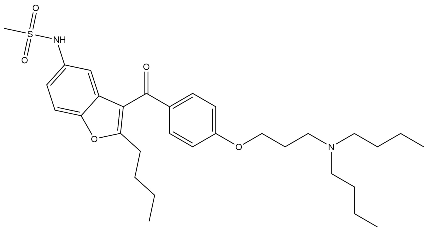
III	Ibutilide	yes	yes	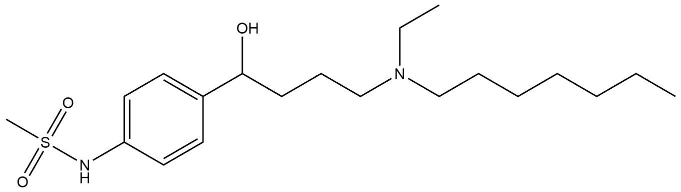
III	Sotalol	yes	yes	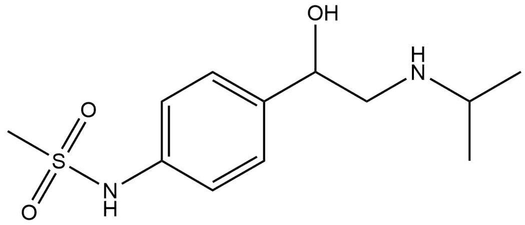
III	Vernakalant	yes	-[3]	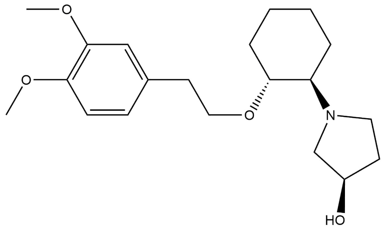
IV	Diltiazem	yes	yes	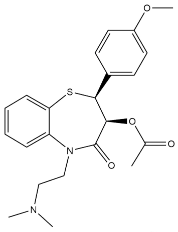
IV	Verapamil	yes	yes	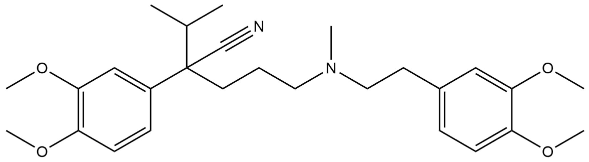

Class	Drug	ESC[1]	USA[2]	Chemical structure
no class	Digoxin	yes	yes	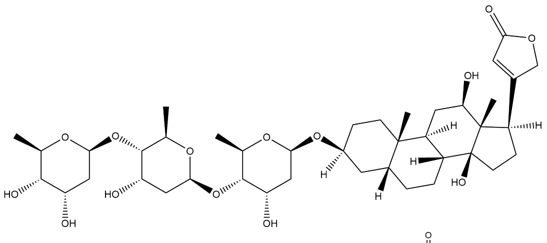
no class	Digitoxin	yes	-[3]	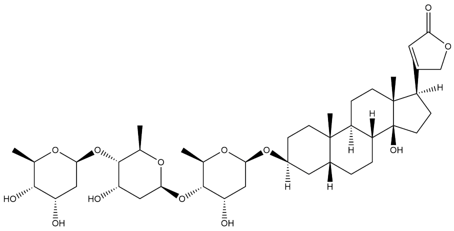

[1] Recommendation by the ESC Guideline 2020 (Hindricks et al., 2020)
[2] Recommendation by the AHA/ACC/HRS Guideline 2014 (January et al., 2014)
[3] Substance not mentioned in the guideline

REFERENCES

Hindricks, G., Potpara, T., Dagres, N., Arbelo, E., Bax, J. J., Blomström-Lundqvist, C., et al. (2020). 2020 ESC Guidelines for the diagnosis and management of atrial fibrillation developed in collaboration with the European Association of Cardio-Thoracic Surgery (EACTS). *Eur Heart J* 00, 1–125. doi:10.1093/eurheartj/ehaa612

January, C. T., Wann, L. S., Alpert, J. S., Calkins, H., Cigarroa, J. E., Cleveland, J. C., et al. (2014). 2014 AHA/ACC/HRS guideline for the management of patients with atrial fibrillation: a report of the American College of Cardiology/American Heart Association Task Force on Practice Guidelines and the Heart Rhythm Society. *J Am Coll Cardiol* 64, e1–e76. doi:10.1016/j.jacc.2014.03.022
